# Practical Notes on Diagnosis and Treatment

**Published:** 1907-07-06

**Authors:** 


### PRACTICAL NOTES ON DIAGNOSIS AND TREATMENT.

**Psoas Abscess and Spinal Disease.**

ALTHOUGH rigidity and other objective signs of caries of the spine nearly always exist with psoas abscess, cases are sometimes met with in which the abscess is the only evidence of spinal disease, all other symptoms being wanting.—*Sir Wm. Bennett.*

**Diet in Aneurysm.**

As regards diet, my own feeling is that you should control the fluids, but not to such an extent as to make the patient uncomfortable. Keep the fluid down to a pint; if less will satisfy without discomfort, give less. Give a generous amount of solids; this, I consider is physiological. But I do not myself see how the starving of people is going to promote coagulation.—*Dr. Goodhart.*

**Abdominal Pulsation in Women.**

IN a nervous woman, the subject of conspicuous abdominal pulsation, the question of aneurysm need hardly be considered. Especially is this true if syphilis can be excluded. Apart from syphilis, women hardly ever suffer from aneurysm, as they are not commonly subjected to the strain to which men are liable.

**Dyspnœa in Functional Disease.**

A PECULIARITY of the rapid respiration which is sometimes seen in functional disease is that the rhythm resumes its normal frequency during sleep. A patient has been known to have a respiratory rate of 140 per minute, and to be distressed and cyanosed during the day, and yet during sleep to have deep and regular respirations numbering only 16 per minute.—*Dr. A. G. Phear.*

**Indigestion.**

THE following is presented as a suggestion for the medicinal treatment of indigestion—℞ Ol. caryoph. ♏ij., acid hydrochlor. dil. ♏xv., tinct. nucis vom. ♏x., tinct. cardomomi co. ʒij. One dose, to be given in water before food. If there is much pain, add a little spirit of chloroform; if acid eructation, replace the hydrochloric acid by 10 to 20 grains of sodium bicarbonate; if constipation, order also liquid extract of cascara sagrada at bedtime.

**The "Blue Line" in Lead Poisoning.**

ALCOHOL, arsenic, and perhaps other metallic poisons may cause the same palsy [as lead], but the absence of a line upon the gums excludes lead as a cause on one condition—provided the gums are anywhere separated from the teeth by a space in which there are decomposing albuminous materials capable of yielding sulphur. With perfect gums you can only exclude lead-poisoning by excluding its possible causes, especially by having the drinking-water analysed and by having the urine analysed after iodide of potassium has been taken for a week.—*Sir Wm. Gowers.*

**Turpentine as a Hæmostatic.**

THE hæmostatic properties of turpentine may be invoked in almost all hæmorrhages from mucous membranes. In bleeding from the gums or from the socket of a tooth, it may be applied locally; otherwise it may be prescribed in the form of an emulsion.

**Gastric Crisis in Diabetes.**

ACCORDING to Dr. Karl Grube, typical gastric crises, exactly similar to those seen in tabes dorsalis, may occur in patients the subject of diabetes. They may last from an hour up to a day or two, and leave great prostration behind them. The best treatment is large enemata to clear the bowel and hot applications to the abdomen. Such crises are of bad prognostic significance.

**Prognosis in Phthisis Pulmonalis.**

Do not make your prognosis so much depend on the extent of the local disease as upon the general symptoms pointing to system poisoning. Always remember the enormous importance of preserving the capacity of taking nourishment in tubercular patients; as soon as this fails the condition becomes one of anxiety.—*Dr. Arthur Hall.*

**Pityriasis Versicolor.**

THE application of a solution of perchloride of mercury (gr. ij. to ℥j.) or of sodium hyposulphite (ʒj. to ℥j.) twice daily, and continued for some time after the eruption has disappeared, is usually successful. Another method is thorough scrubbing in a bath with the following: Hydrarg. perchlor., gr. xx.; saponis viridis, ℥iij.; rectified spirit, ℥ij.; oil of lavender, ♏xx.—*Sir Thos. McCall Anderson.*

**The Passage of a Sound.**

IN middle-aged and elderly men coming under the category of those needing the use of a sound, this should be employed with all the precautions and resources that a comfortable bed, an anæsthetic, antiseptics, and proper precautions afford. If these conditions are not satisfied, the patient runs the risk of such consequences as rigors, high temperatures, a swelled testicle, or cystitis.—*Mr. Reginald Harrison.*

**Boric Acid Poultice.**

MIX a tablespoonful of cold water starch and a teaspoonful of boric acid with a little water; add the mixture to a pint of boiling water and stir the whole until a uniform mucilaginous mass is formed. When cold spread the jelly thickly on cotton and cover it with a piece of muslin. Then apply to the part. A good plan is to put on the poultice at bedtime and to remove it in the morning. It is useful in acute and subacute skin affections to cleanse and soothe prior to the application of ointments, etc.

